# Fulminant Supraglottitis from *Neisseria meningitidis*

**DOI:** 10.3201/eid1303.061420

**Published:** 2007-03

**Authors:** Mark G. Kortepeter, Brian L. Adams, Wendell D. Zollinger, Robert A. Gasser

**Affiliations:** * Walter Reed Army Medical Center, Washington, DC, USA; † Madigan Army Medical Center, Fort Lewis, Washington, USA; ‡ Walter Reed Army Institute of Research, Silver Spring, Maryland, USA

**Keywords:** *Neisseria meningitidis*, meningococcus, neck infection, meningococcal supraglottitis, supraglottitis, epiglottitis

**To the Editor:** A 68-year-old Caucasian woman with non–insulin-dependent diabetes mellitus, hypertension, and peripheral vascular disease sought treatment at an emergency department after experiencing 2 days of pharyngitis and 1 day of fatigue and dysphagia for solid food. The morning of admission she noted dysphagia for solid food and liquids, dysphonia, severe anterior neck pain, swelling, and erythema, dyspnea, and a temperature of 102.3°F (39°C). A computed tomographic (CT) scan demonstrated substantial neck soft tissue edema and narrowing of the oropharynx and hypopharynx. She received single doses of intravenous ampicillin/sulbactam, clindamycin, dexamethasone (10 mg), and methylprednisolone (125 mg) before being evacuated by air to our intensive care unit (ICU) at Walter Reed Army Medical Center. Intravenous ampicillin/sulbactam, 3 g every 6 hours, and clindamycin, 900 mg every 8 hours, were continued after the transfer. Two doses of intravenous vancomycin, 1 g every 12 hours, were given before vancomycin was discontinued. Results of laboratory studies were the following: leukocyte count 13.3/mm^3^ (71% polymorphonuclear leukocytes, 18% bands) and normal hematocrit, platelet count, blood urea nitrogen and creatinine concentrations, and liver-associated enzymes.

A marker pen was used to track the rapid advance of erythema overnight from her anterior, inferior chin to the top of her breasts (Figure). The infectious disease service was consulted the next morning. When she was examined, her condition had improved; she had normal vital signs, a slightly hoarse voice, and the ability to swallow some saliva. She had no headache or meningismus. The chest erythema was receding. Oral examination demonstrated erythema and an abrasion in the posterior pharynx. Her tongue was not elevated and her uvula was midline. Anterior firm edema without crepitus extended from her chin to the mid-neck. Results of her examination were otherwise unremarkable. The infectious disease consultant recommended restarting a course of vancomycin and discontinuing clindamycin.

**Figure:**
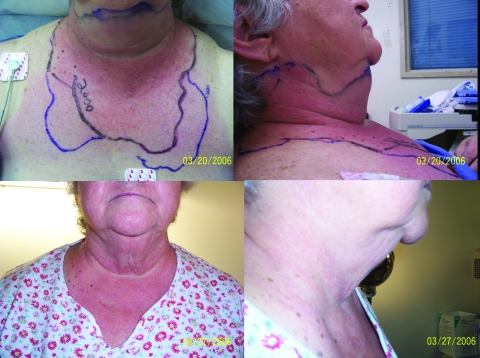
Top, anterior and lateral views of patient on day 1 of receiving antimicrobial drugs, demonstrating neck erythema and edema. Bottom, anterior and lateral views of patient on day 8 of receiving antimicrobial drugs, demonstrating resolution of neck erythema and edema.

A follow-up CT scan with contrast demonstrated anterior cervical soft tissue edema and patent airway with surrounding abnormal thickness and soft tissue density. No abscess or clot was seen. Endoscopic examination in the ICU showed diffuse erythema and generalized supraglottic edema affecting mostly the epiglottis and arytenoids. Dental examination demonstrated no acute pathologic features. Blood cultures at our hospital yielded no growth, and throat culture was negative for group A streptococci.

The patient recovered without requiring intubation (Figure). On the day of discharge, a blood culture from the referring hospital’s emergency department was reported to be positive for *Neisseria meningitidis*, serogroup Y. Immediate family members and the otolaryngologists who conducted the endoscopic examination were given postexposure prophylaxis. The patient also received terminal prophylaxis. Results of a screening CH50 for terminal complement deficiency were normal.

This patient’s condition is consistent with fulminant meningococcal supraglottitis. Supraglottitis implies involvement of the epiglottis and surrounding structures and is more commonly used to describe adult infection than is epiglottitis (*1*). Epiglottitis has become more common in adults than in children since the introduction of the *Haemophilus influenzae* type b vaccine. Other organisms responsible for epiglottitis in adults include *H. influenzae*, *H. parainfluenzae*, pneumococci, *Staphylococcus aureus*, and group A streptococci (*2*).

Despite its propensity to colonize the upper respiratory tract, *N. meningitidis* has rarely been identified as a cause of supraglottitis or other deep neck infections. Only 6 cases have been reported, the first in 1995 (*3*–*8*). Previously reported cases were equally apportioned by sex, and patients were 44 to 95 years of age (*3*–*8*). Including our patient, 3 of 7 were diabetic (*6*,*7*). None showed evidence of meningitis or fulminant meningococcemia, but all had fever, pharyngitis, and airway compromise. Five required airway intervention: 3 intubations and 2 urgent tracheostomies. Two received steroids (*3*,*4*), a 54-year-old man required urgent tracheostomy before receiving steroids, and a 60-year-old man’s condition “deteriorated rapidly,” but the report does not indicate the interval between receipt of steroids and intubation. Although steroids have been used, their benefit is unproven, and no controlled clinical trials have been conducted (*9*).

Blood cultures have been positive from all reported case-patients. Two isolates were typed as serogroup B, 4 as serogroup Y, and the serotype of 1 was unreported. Meningococcal strains causing supraglottitis appear to be more locally aggressive but cause less disseminated disease, possibly due to decreased tropism for endothelial cells (*8*).

To our knowledge, ours is the second case of meningococcal supraglottitis reported with severe neck edema and cellulitis; a 44-year-old woman in a prior review had features similar to our patient (*8*). An 81-year-old woman with diabetes was noted to have “reddish swelling” on the right side of the neck (*7*), but little was described beyond that. All 3 had serogroup Y infection. We wondered whether serogroup Y might have a propensity to cause cellulitis; however, a review of 10 cases of meningococcal cellulitis included patients with multiple serogroups: C (4 cases), B (2 cases), Y (2 cases), and unknown (2 cases) (*10*).

*N. meningitidis* may cause supraglottitis more frequently than is recognized (*3*). Timely drawing of blood cultures in relation to administration of antimicrobial drugs is most likely to identify this pathogen in this setting. Because of its public health implications and potential for rapid progression to airway compromise, *N. meningitidis* should be considered among the differential diagnoses of supraglottitis/epiglottitis.
